# Analysing the significance of small conformational changes and low occupancy states in serial crystallographic data

**DOI:** 10.1002/2211-5463.70218

**Published:** 2026-03-11

**Authors:** Jake Hill, Yelyzaveta Pulnova, Elke De Zitter, Helen M. Ginn, Briony A. Yorke

**Affiliations:** ^1^ School of Biomedical Science University of Leeds UK; ^2^ ELI Beamlines Facility Extreme Light Infrastructure (ERIC) Dolni Brezany Czechia; ^3^ Faculty of Mathematics and Physics Charles University Prague Czechia; ^4^ Univ. Grenoble Alpes, CNRS, CEA Institut de Biologie Structurale France; ^5^ Center for Free‐Electron Laser Science CFEL Deutsches Elektronen‐Synchrotron DESY Hamburg Germany; ^6^ Institute for Nanostructure and Solid State Physics University of Hamburg Germany; ^7^ The Hamburg Centre for Ultrafast Imaging Germany; ^8^ School of Chemistry and Astbury Centre University of Leeds UK

**Keywords:** crystallographic software, structural biology, time‐resolved crystallography

## Abstract

The interpretation of electron density maps from time‐resolved serial diffraction experiments is often hindered by incomplete initiation and mixtures of states. Additionally, it can be challenging to determine the significance of small conformational changes. Here, we present a protocol that exploits the inherent oversampling of serial crystallographic data through batch resampling and principal component analysis (PCA). This approach provides insight into the significance of small conformational changes in proteins along a reaction time course. When combined with extrapolation of structure factor amplitudes, the method further helps in the identification of low‐occupancy intermediates. In this protocol report, we describe a practical workflow for batch resampling, scaling and clustering, and provide guidance for the effective use of open‐source software including *RoPE* and *Xtrapol8*.

## Graphical abstract

This protocol paper outlines methods to establish the success of a time‐resolved serial crystallographic experiment, by means of statistical analysis of timepoint data in reciprocal space and models in real space. We show how to amplify the signal from excited states to visualise structural changes in successful experiments.

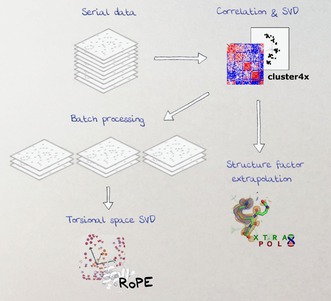

**Abbreviations**
CBF: crystallographic binary format
CC: correlation coefficient
CCP4: Collaborative Computational Project No. 4
CIF: crystallographic information file
ESFA: extrapolated structure factor amplitude
GUI: graphical user interface
PCA: principal component analysis
PDB: protein Data Bank file
SVD: singular value decomposition
TRSX: time‐resolved serial X‐ray crystallography

## Introduction

**Fig. 1:**
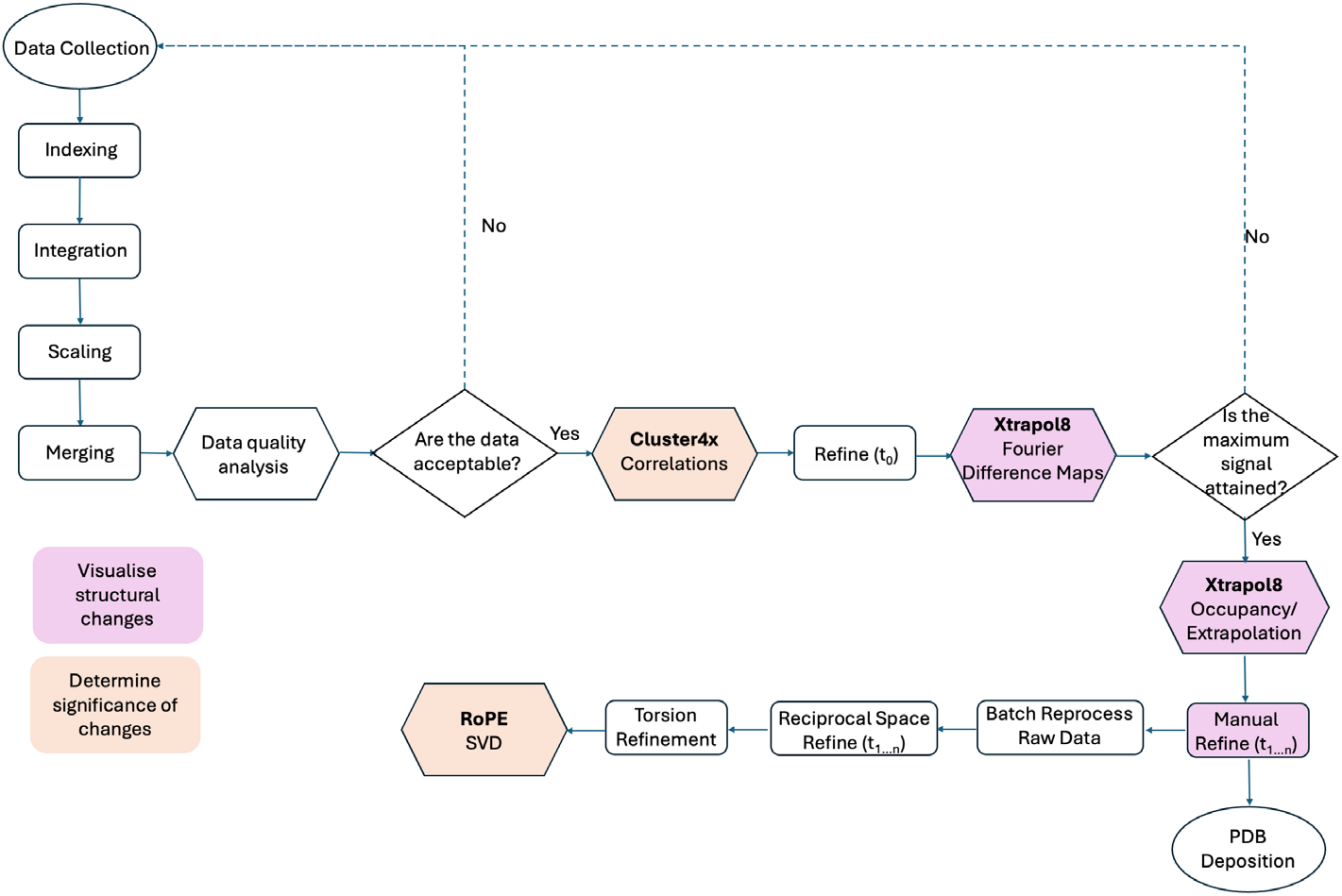
Flowchart showing the recommended data analysis workflow. Ovals represent the beginning and end points. Rectangles represent data processing steps. Hexagons are analysis points and diamonds represent decisions.

Time‐resolved serial X‐ray crystallography (TRSX) allows the direct observation of protein dynamics and function in real time. TRSX has been used to characterise a wide range of light‐activated biological processes across timescales ranging from femtoseconds to minutes [1]. Applications include the characterisation of electronically excited states (e.g. flavin adenine dinucleotide in photolyase [2, 3]), the identification of previously unknown intermediate conformations (e.g. photoactive yellow protein [4], bacteriorhodopsin [5]) and the elucidation of allosteric transitions (e.g. fluoroacetate dehalogenase [6]). Despite its transformative potential, interpretation of electron density maps in TRSX remains challenging. Data are often complicated by incomplete photoinitiation, the presence of multiple intermediate states, and poorly defined geometries of excited states. Careful experimental design, supported by complementary spectroscopies (including UV/Vis and Raman) and computational methods (molecular dynamics, quantum mechanics simulations), can reduce ambiguity. However, the detection and reliable characterisation of small but significant structural changes and low‐occupancy states remains particularly difficult. Time‐resolved experiments are technically challenging. There are many individual components that must work together in order to result in a successful experiment (e.g., initiation—laser alignment and timing, sample quality). Many experiments may lack signal in consequence of any component failing. Visual inspection of electron density is prone to confirmation bias and should not be entirely relied upon to provide evidence of success. Here, we present a practical guide to exploit the inherent oversampling in serial crystallography datasets where subtle structural changes are expected. This protocol aids in the identification of failed experiments where the null hypothesis holds and also in the interpretation of data from successful experiments. The workflow (Fig. 1) integrates conventional serial crystallography pipelines with batch resampling and downstream analysis in reciprocal, torsion angle and real space. Many of the methods described in this protocol are still under active development. We recommend that readers also familiarise themselves with alternative approaches [7] including denoising [8], singular value decomposition [9] and multi‐dataset background reduction [10], to name a few.

## Materials

### Software and tools

- *DIALS* v3.10 or later [11].
- *CrystFEL* v0.11 or later [12].
- *Vagabond* (includes *cluster4x* for SVD and *shell_scale* for resolution‐dependent scaling [13])
- *RoPE* v0.1.4 or later (torsional refinement and PCA [14]).
- *Xtrapol8* v1.2.6 (structure factor amplitude extrapolation [15]).
- Refinement software: *ccp4* v9.0 [16] (including *REFMAC5* v5.5 [17], *ServalCat* [18]) and phenix v1.21 [19].
- *MatchMaps* v0.7.3 [20].
- *Python* v3.8+ with *numpy*, *scipy*, and *pandas*.

### Hardware

- Sufficient CPU cores (≥ 32 recommended for *CrystFEL* batch jobs).
- GPU acceleration is recommended for fast peak finding.
- High‐performance computing cluster with Slurm or equivalent job scheduler recommended.

### Time‐resolved data

- Time‐resolved datasets—including a reference set of the dark of untriggered state ($t_{0}$) and at least one triggered set, which is collected at a specific time delay after reaction initiation in the crystals. Diffraction images should be in NeXus format [21] or Crystallographic Binary Format (CBF).
- Merged complete and half datasets in MTZ format.

## Methods

### On‐the‐fly data processing

#### Data reduction and quality assessment

During serial crystallography data collection, the reference dataset ($t_{0}$) and sets collected at specific time delays ($t_{1}\ldots t_{n}$, timepoints) after a reaction triggering event should be processed using standard serial crystallography pipelines (e.g., *xia2.ssx* with *DIALS* [11] and *CrystFEL* [12]). This protocol focusses on the analysis of reduced data, that is after successful indexing, integration, scaling and merging. These prior steps are covered in detail elsewhere [22, 23, 24]. However, it is important to note that high‐quality data are essential when attempting to identify low‐occupancy states. During data collection, optimise the data quality indicators according to the categories below before using them to assess any experimental adjustments that need to be made.

#### Spot‐finding and hit rate

##### Optimise

Spot‐finding parameters are available in both *CrystFEL* and *DIALS* and generally allow the user to change the minimum pixel count in a putative peak or the peak/background thresholds. Optimal parameters will pick up spots only attributable to diffraction, whereas clusters of spots, especially those which are common to all images, should be avoided or masked. Appropriate peak/background threshold values are dependent on the type of detector and beamline used. Visual inspection of diffraction images can help to determine a suitable threshold value when using a new detector. In the *DIALS* image viewer, *dials.image_viewer*, spot‐finding parameters can be changed manually and the effects of adjustments can be seen by displaying the threshold view of the image [11]. In *CrystFEL*, the *CrystFEL* graphical user interface (GUI) can be used to view images and determine a suitable threshold [12].

##### Assessment

Use the hit rate to inform the experimental design, for example, is sample delivery neither too sparse nor too crowded. For high hit‐rates (e.g., > 50%), the frequency of multiple lattices in a single image increases. Multiple lattices originate from overlapping crystals. In this case, consider the laser penetration depth and crystal thickness to decide whether individual lattices may represent the untriggered state. In many cases, only the top crystal may be triggered and currently no method exists to identify the triggered/untriggered lattices in an image.

#### Indexing

For time‐resolved studies, maintaining consistency in indexing behaviour across time points is crucial to avoid introducing artificial differences. This means storing samples under unfluctuating environmental conditions, maintaining the detector geometry set‐up established at the beginning of the experiment, and completing an experiment within a single beamtime.

##### Optimise

- Unit cell parameters should be determined prior to TRSX beamtime. Micro‐adjustments may be required during beamtime to account for sample behaviour. In *CrystFEL* *indexamajig*, use the ‐p option to supply a reference unit cell either from previous runs, or fitted from initial indexing.
- Samples may comprise naturally heterogeneous crystals without a tight distribution of unit cells. In *indexamajig*, the ‐‐tolerance parameter is critical for preventing merging of nonisomorphous data. Set axis tolerances according to expected unit cell variability (e.g. dehydration effects, temperature gradients, or ligand‐induced strain). Example: ‐‐tolerance 5, 10, 5, 1.5 tolerates higher variation in the *b*‐axis than the default setting (5, 5, 5, 1.5).
- Always enable multiple lattice indexing, in *CrystFEL* *indexamajig*, ‐‐multi or for *xia2.ssx* in *DIALS* max_lattices = n, where n>1. When the penetration depth of the initiating laser is limited, overlapping crystals will result in a mixture of states. For all timepoint datasets ($t_{1}\ldots t_{n}$), images with multiple lattices can be discarded where appropriate. For the reference state $t_{0}$, multiple lattice indexing will increase the number of indexed patterns and identify overlapping reflections that are excluded from further analysis.
- Samples chosen for time‐resolved experiments must be of sufficiently high resolution to support determination of time‐resolved structural changes. However, detector distance must be calibrated to avoid being ‘greedy’ for high resolution beyond where the crystals can diffract, as this concentrates the background counts and leads to poorer estimation of intensities. Conversely, ordered reflections at high resolution should not be cut due to a detector pushed too far back.
- Detector geometry should be refined from an up‐to‐date geometry file (e.g. using the *Millepede‐II* implementation in *CrystFEL*, doi:10.5281/zenodo.13904047 or *dials.refine* in *DIALS* [25]). In the majority of experiments, geometry refinement should be performed if the detector, beam or sample alignment has physically changed. In this case, it should be limited to rigid body refinement of the entire detector. Internal panel movements should typically only be refined when the detector has been dismantled and rebuilt. Refinement should be performed against strong, high‐quality data from isomorphous crystals with high, ideally cubic symmetry (e.g. cubic insulin). Lysozyme is a common choice for detector geometry refinement due to its ease of crystallisation and handling. One significant downside is the unit cell dimensions are typically heterogeneous and sensitive to humidity, and therefore may not the best choice for this purpose especially if other options are available.

The indexing rate will be dependent on the quality of the spot‐finding, accuracy of the geometry file and unit cell dimensions. These are worth optimising during beamtime as the projected number of indexed patterns will directly inform the extent of time points that can be explored.

##### Assessment

- Indexed unit cell: During indexing, unit cell dimensions are adjusted to improve the fit to the diffraction pattern. When the modelled geometry differs significantly from reality, or due to systematic errors, the spread of refined unit cell dimensions tends to inflate. Therefore, assessing tightness of the distribution is helpful for diagnosing indexing problems. This can be done using *CrystFEL cell_explorer* or *dials.cluster_unit_cell* in *DIALS*.
- Number of indexed patterns: A minimum of ≈ 5000 indexable patterns is thought to support a basic structure solution [22], although this is also dependent on the number of crystal symmetry operations and data quality. In order to support successful determination of smaller amplitude differences, this may increase by an order of magnitude or more. We advise that highly redundant data are collected to support the analysis of small conformational changes and low occupancy states. Therefore, indexing rates determined during beamtime will guide the number of timepoints chosen, with consideration of the time and sample available. Timepoints should be collected in order of scientific value.

#### Integration, scaling and merging

At this stage, assessment of data quality indicators can further guide the experimental procedure.

##### Assessment

- Signal‐to‐noise: Too low signal strength at high resolutions may alter proper scaling and estimation of Wilson B factors. This will result in apparent low B factors in structures refined in extrapolated structure factor amplitudes (ESFAs). This may potentially indicate a ‘greedy’ detector distance has been used and it should be pushed further back, but individual images should be inspected for high‐resolution spots in case this is actually the result of an indexing problem (see above).
- Completeness: A bare minimum of 95% is needed to support a basic structure solution [23, 26], which will fall short of what is required for TRSX. In TRSX, the main target is to bring multiplicity into the tens or hundreds of observations per reflection. However, the measure of ‘completeness’ is only sensitive to whether the number of observations ≥1. If the overall multiplicity is so low that 100% completeness is in question, then the processed dataset will likely be of insufficient quality to support TRSX, primarily due to low multiplicity. However, monitoring completeness is still useful as it can provide an early indication of problematic preferred orientations by the sample if the multiplicity is simultaneously high. This is hard to address during beamtime but may be partially mitigated, for example by collecting diffraction from loaded chips at an angle to the beam.
- Multiplicity: High multiplicity is necessary to ensure robust scaling and error estimation [22, 27] and allows for binning and subsequent clustering and singular value decomposition. After enough patterns have been indexed that scaling is stable, a doubling of the multiplicity will improve the precision of the intensity estimation by $\sqrt{2}$. This means that an interim dataset with a known number of contributing patterns can be used to predict the likely number of patterns needed to achieve a certain quality.
- $R_{\text{split}}$: A commonly used indicator in serial crystallography [12] and roughly equivalent to $R_{\mathrm{pim}}$ [28] but only requires calculation of the half‐datasets rather than retaining each individual observation. This is an indicator which will eventually improve by $\sqrt{N}$ for an N‐fold increase in patterns.
- ${\mathrm{CC}}_{1/2}$: The half correlation coefficient (CC) increases with number of indexable images. Although this appears to flatten beyond a threshold number of patterns, the correlation still exhibits a logarithmic improvement towards 100%. Monitoring of the highest resolution shells can be more sensitive to whether more data are required and inform potential resolution cut‐offs. Determining a suitable resolution cut‐off is discussed in greater depth elsewhere [29, 30, 31].

Each dataset (especially the reference, $t_{0}$) should be satisfactory before moving onto the next.

#### Preliminary assessment of structural changes

Differences between the datasets collected for the dark or reference state before initiation ($t_{0}$) and at each timepoint ($t_{1}\ldots t_{n}$) are monitored on‐the‐fly to confirm that reaction initiation has been successful and that structural changes have been triggered. This analysis is performed in two ways: (a) Correlation matrices generated from structure factor amplitudes to highlight global changes in reciprocal space. (b) Fourier difference electron density maps are calculated to highlight and monitor site‐specific structural changes in real space. The latter approach was, for example, used in Rios *et al*. [32], to determine the presence of structural changes and also to ascertain if the minimum number of indexed patterns required to generate high‐quality maps was collected.

Both approaches require MTZ files containing structure factor amplitudes. When calculating correlation matrices, use half‐datasets from each timepoint to verify that there is a strong correlation within timepoints and that subsequent time‐resolved changes are highlighted by a decrease in the correlation as the reaction proceeds. Half‐datasets are automatically generated by *CrystFEL* during scaling and postrefinement of partial reflections using *partialator*.

#### Correlation matrices in reciprocal space

The *cluster4x* subpackage in the *Vagabond* package is used to generate correlation matrices by using Pearson calculated between the series of structure factor amplitudes relative to the mean in each dataset ($t_{0}\ldots t_{n}$) [13]. Alternative clustering algorithms are available (e.g. in *XDS* [33], *xia2multiplex* in *DIALS* [34], *blend* in *Collaborative Computational Project No. 4* (*CCP4*) [35, 36]). In contrast to other methods, *cluster4x* highlights the consistency of amplitude differences relative to the mean as opposed to absolute values. To do this, first generate a list of MTZ files for the reference and all timepoint half‐datasets, for example when all files are in the same directory:


 $ ls -d $PWD/*.mtz > mtz_files.txt





**Fig. 2:**
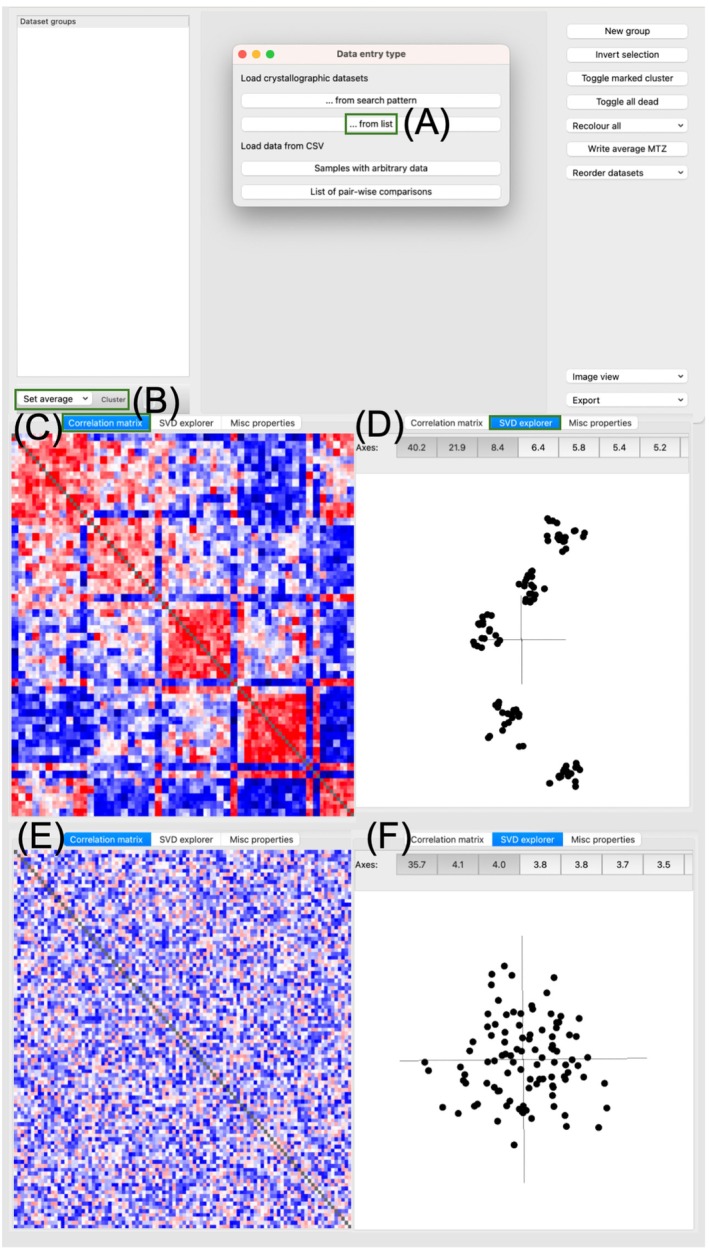
*cluster4x* GUI (A) load crystallographic datasets (MTZ format) from list. (B) click cluster to generate average datasets from structure factor amplitudes, calculate Pearson correlation coefficients and perform SVD. (C) this generates a correlation matrix. In the correlation matrix, the colour of element *A*_*ij*_ corresponds to the Pearson correlation coefficient between dataset *i* and dataset *j*. The colour scale ranges from blue (coefficient of 0) through white (coefficient of 0.5) to red (coefficient of 1). An example matrix is shown for a successful experiment. (D) click SVD to switch to plot view. Plot shows first and second principal axes for the SVD of the correlation matrix in (C). (E) similar correlation matrix in the case of noise (unsuccessful experiment). (F) plot showing first and second principal axes for SVD of the unsuccessful experiment. User‐selectable elements are indicated by green rectangles. Data (*n* = 50) generated for illustrative purposes only.

Then in the *cluster4x* GUI (Fig. 2):

1. Load crystallographic datasets *from list* (mtz_files.txt). During this step, all datasets are automatically scaled using the *shell_scale* algorithm [13].
2. Select *Use amplitudes* from the drop‐down *Set average* menu. Then click *cluster*. Average datasets are then generated in reciprocal space. Pairwise correlation coefficients are calculated between pairs of datasets relative to the average and singular value decomposition performed on the corresponding correlation matrix.
3. The *Correlation matrix* tab shows a 2D correlation plot representing the relationships between datasets in reciprocal space.
4. The *singular value decomposition* (*SVD*) tab shows a 3D plot of each dataset projecting the top 3 principal axes. Alternative principal axes can be selected by clicking the *Axes* tabs.
5. Half‐datasets from the same timepoint should be strongly correlated and closely clustered, whereas time‐resolved structural changes can manifest systematic shifts along one or more principal axes. Largely, diffraction from different timepoints do not tend to correlate with one another except due to noise, except for when the timepoints have approximately femtosecond‐scale separations, at which point neighbouring timepoints may correlate. Distinct clusters in this space indicate differences in the underlying electron density features, which may correspond to conformational changes, ligand binding or reaction intermediates. Figure 2 highlights the differences between data that successfully clusters (Steps 3 and 4) and data that shows only random noise between timepoints (Steps 5 and 6) indicating that the experiment may have failed.

Outliers in the correlation matrix and SVD plot can highlight problematic datasets or unexpected states that merit further inspection.

#### Fourier difference ($F_{\mathrm{obs}}$ − $F_{\mathrm{obs}}$) electron density maps

Difference maps highlighting structural differences between a reference dataset and that for a particular timepoint, that is $F_{\mathrm{obs}}^{t_{i}}-F_{\mathrm{obs}}^{t_{0}}$ are calculated. To do this:

1. Refine a model against the reference dataset (t_0.mtz) to provide reference phases (t_0.pdb).
2. Scale the reference ($t_{0}$) and triggered ($t_{i}$) structure factor amplitudes. As reaction initiation often introduces disorder it is sensible to use $t_{0}$ as the scaling reference. The *shell_scale* protocol from *Vagabond* can be executed from the command line:


 $ shell_scale path/to/t_0.mtz path/to/t_i.mtz

A new MTZ file is created in the same directory with the prefix shsc‐ and scaled structure factor amplitudes are written to the column FP.

3 Calculate the $F_{\mathrm{obs}}^{t_{i}}-F_{\mathrm{obs}}^{t_{0}}$ difference map using phases from the reference state model.

This can be performed using Phenix to output an fi_minus_f0.mtz file that can be loaded as a difference map in Coot [37] for inspection.


$ phenix.fobs_minus_fobs_map f_obs_1_file=shsc-t_i.mtz


f_obs_2_file=shsc-t_0.mtz phase_source=t_0.pdb high_res=2.0


sigma_cutoff=2 scattering_table=n_gaussian





The same can be achieved using *Xtrapol8* in $F_{o}-F_{o}$ only mode:


$ phenix.python <folder/to/Xtrapol8>/Fextr.py X8.phil





Using the X8.phil file:


input {


   reference_mtz = t_0.mtz


   triggered_mtz = shsc-t_i.mtz


   reference_pdb = t_0.pdb


}


scaling {


  b_scaling = no #if data are already scaled, e.g. with shell_scale or


     partialator


  #b_scaling = anisotropic #if data is not yet scaled


}


f_and_maps {


  fofo_type =  qfofo


}


output {


  outdir = ti_minus_t0


  generate_fofo_only = True


}





**Fig. 3:**
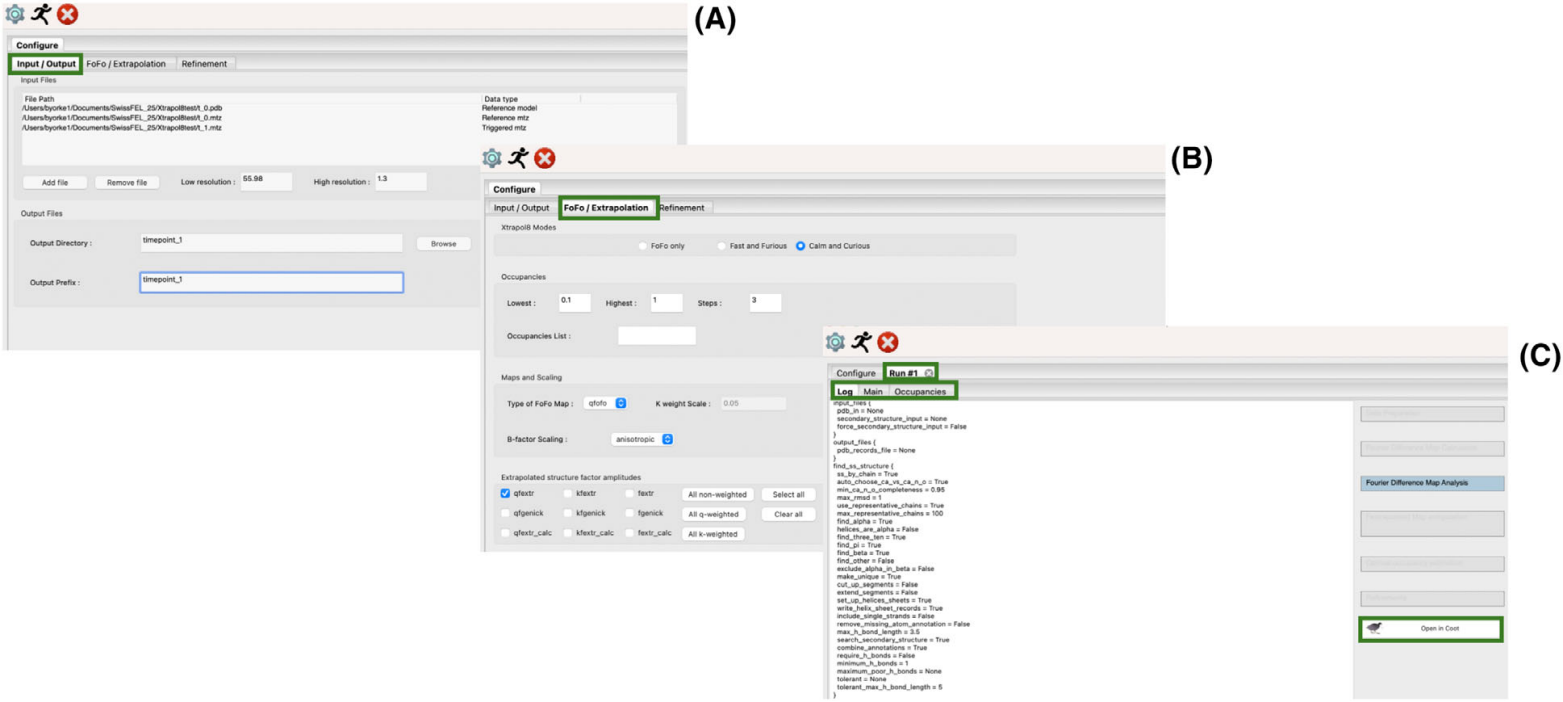
*Xtrapol8* GUI. (A) Reference and triggered‐state data and reference model are loaded in the *Input/Output* tab. (B) The *FoFo/Extrapolation* tab is used to choose the running mode and allows selection of different Bayesian weighting schemes. In a third tab *Refinement* (not shown), options for reciprocal and real space refinement can be chosen. (C) Output is provided per run and consists of a log file, output graphs, and a button to open results directly in Coot. User‐selectable elements are indicated by green rectangles.

This can also be accomplished in the *Xtrapol8* GUI, in which the reference model in either Protein Data Bank file (PDB) or Crystallographic Information File (CIF) format, reference dataset (MTZ or CIF format) and triggered dataset (MTZ or CIF format) can be loaded. Afterwards select *FoFo only* in the *FoFo/Extrapolation* tab (Fig. 3) followed by pressing *Run*. A comprehensive list of input parameters for *Xtrapol8* can be found at https://www.github.com/ElkeDeZitter/Xtrapol8/. The key outputs are the $F_{\mathrm{obs}}^{t_{i}}-F_{\mathrm{obs}}^{t_{0}}$ map in *ccp4* map and MTZ formats, as well as a comprehensive log file, a script for loading the maps and models directly into *COOT*, and a number of figures indicating the isomorphism between the dataset and localisation of the peaks in the Fourier difference map.

Scaling to the reference dataset can also be performed within *Xtrapol8*, which will use the *ccp4* *SCALEIT* program with isotropic or anisotropic B‐factor modelling ($b_{\text{scaling}}$ parameter). Phenix also implements an alternative scaling protocol when calculating difference maps. Thus, the *shell_scale* step may be skipped in both cases. However, it may be necessary to try different scaling and weighting approaches to obtain the clearest map.

Importantly, isomorphism between the reference and triggered datasets is calculated as both an R‐factor ($R_{\mathrm{iso}}$) and correlation coefficient (${\mathrm{CC}}_{\mathrm{iso}}$). These values, alongside the plot of $R_{\mathrm{iso}}$ and ${\mathrm{CC}}_{\mathrm{iso}}$ against resolution can be used to assess whether the difference maps and downstream calculation of ESFAs are reliable. Where data are nonisomorphous ($R_{\mathrm{iso}}>>0.10$ overall or $R_{\mathrm{iso}}>0.35$ for the highest resolution shell), the phases from the reference state model may not be compatible with the triggered state structure factor amplitudes and the map may be highly dominated by noise. It is imperative to refrain from relying only on statistical values such as $R_{\mathrm{iso}}$ and all maps should always be inspected visually. On the other hand, a danger of visual inspection alone is overinterpretation of noise at the site of dynamic interest. During visual inspection, regions away from suspected dynamic features should be used as a guide to determine the level of background noise to try to minimise confirmation bias. As a consequence, Fourier difference maps with low isomorphism can still show relevant features if the differences are strong enough to appear above the noise; the key is that the phases are still compatible. Concurrently, maps with high isomorphism can show no relevant peaks if the signal is too weak (e.g. if not enough data are acquired).

Where there are large structural changes that result in nonisomorphous datasets (e.g. a large change in unit cell dimensions between $t_{0}$ and $t_{i}$ datasets or rotation of the entire molecule in the unit cell) *MatchMaps* can be used [20], which will refine each state separately and then calculate the difference map in real space:


$ matchmaps --mtzoff t_0.mtz FP SIGF --mtzon shsc-t_i.mtz FP SIGF


--pdboff t_0.pdb





Care must be taken to ensure the correct headers are provided for the structure factor (FP) and structure factor standard deviation (SIGF) columns. *MatchMaps* applies a solvent mask, outputs a difference map (shsc‐t_i_minus_shsc‐t_0.map), an unmasked difference map (shsc‐t_i_minus_shsc‐t_0_unmasked.map) as well as both aligned (t_i.map / t_0.map) and unaligned (t_i_before.map/t_0_before.map) versions of the separate $t_{0}$ and $t_{i}$ maps.

### Modelling low occupancy states and analysing significance of small structural changes

#### Structure factor amplitude extrapolation

**Fig. 4:**
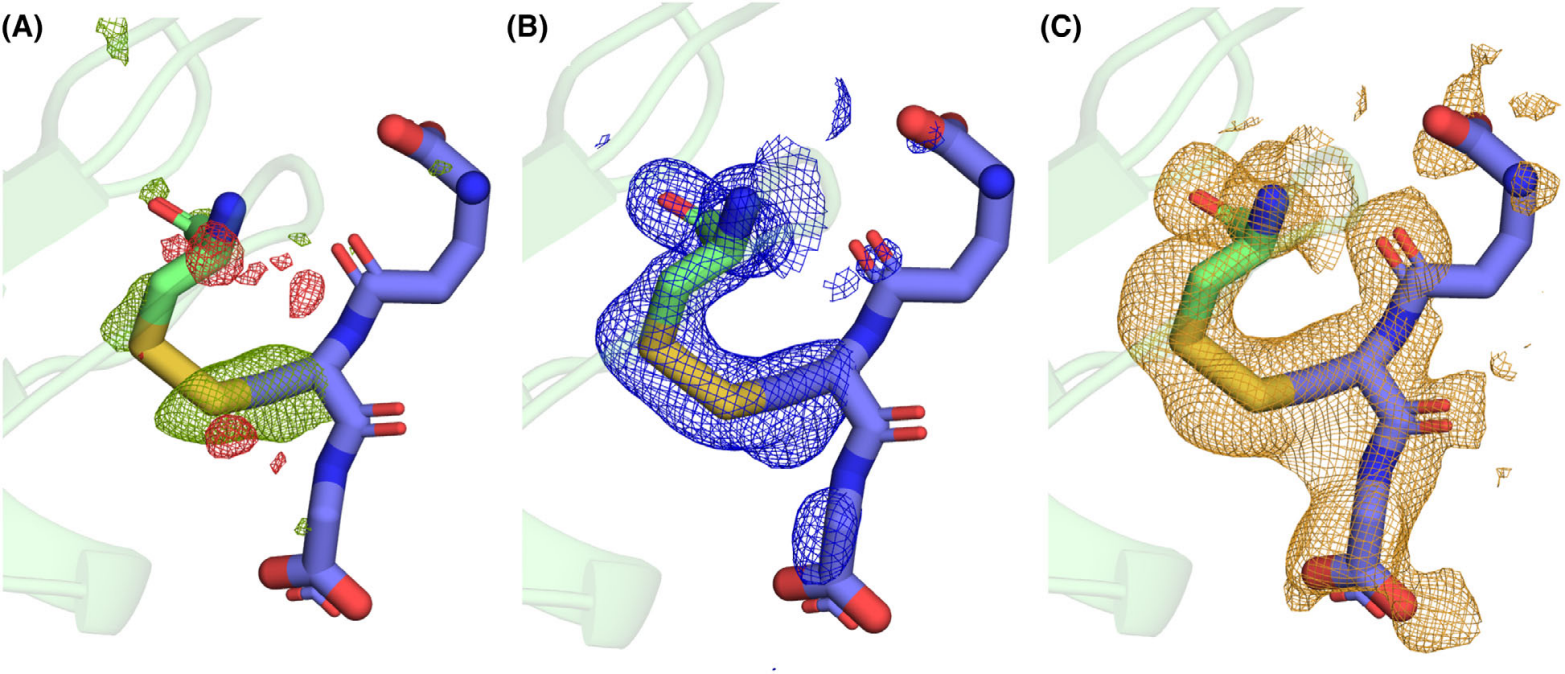
Example of density improvement after calculation of ESFAs. Data collected from human gamma‐D crystallin (shown as cartoon) before (*t*_0_) and after (*t*_1_) formation of glutathione adduct to cysteine (shown as sticks). (A) Fo‐Fo map calculated by subtracting *t*_0_ from *t*_1_, contoured at 3 r.m.s.d and carved at 2.0 Å. (B) original 2mFo‐DFc map in blue, contoured at 1 r.m.s.d and carved at 2.0 Å. (C) extrapolated map 2mF_extrapolated_‐DFc generated by *Xtrapol8* in orange contoured at 1 r.m.s.d and carved at 2.0 Å.

In TRSX studies, incomplete activation of the triggered state is common. This results in multiple partial occupancy states within a dataset, with most often the reference state dominating the signal. The use of ESFAs that model a theoretical 100% occupancy triggered state can help in modelling the triggered state and reveal subtle changes between $t_{0}$ and $t_{i}$. *Xtrapol8* [15] is used to calculate ESFAs with various Bayesian weighting factors. It will also estimate the extrapolation factor, which is related to the occupancy of the triggered state. If multiple triggered states are present in the model, this extrapolation factor may be inaccurate. To calculate ESFAs and estimate occupancy using *Xtrapol8* (Fig. 3):

1. Run *Xtrapol8* in *fast‐and‐furious* mode to obtain a first set of ESFAs and to obtain a first rough estimate of the occupancy based on the peaks in the difference map. The prerequisite is that the Fourier difference map calculated in the previous section must show clear features that can be distinguished from noise. Verify the generated extrapolated electron density map (2 m$F_{\text{extrapolated}}$‐D$F_{\text{calc}}$ and m$F_{\text{extrapolated}}$‐D$F_{\text{calc}}$), paying special attention to their interpretability and if the reference state is still present. The latter two aspects may indicate improper occupancy estimation. If necessary, adapt input parameters to widen or narrow down the range of tested occupancies and alter the parameters for analysis of the electron density maps.
2. Run *Xtrapol8* in the *calm‐and‐curious* mode if proper parameters are found. This will further optimise weighting, input parameters and Bayesian weighting schemes. Running in this mode also permits application of an orthogonal occupancy estimation method, which is based on the comparison of the refined models with distinct occupancies. Alternatively, if the peaks in the Fourier difference map are minimal or if the difference map method does not yield a proper occupancy estimation, then run in *calm‐and‐curious* mode without first optimising a first set of parameters in the *fast‐and‐furious* mode.
3. Run *Xtrapol8* with automated refinements. The triggered model can be manually built into the extrapolated map (Fig. 4). The Bayesian‐weighted ESFAs calculated with the best extrapolation factor can then be used as input for structure refinement of models for each timepoint $t_{1}\ldots t_{n}$.

### Clustering analysis

Once datasets of satisfactory quality have been obtained and changes are apparent in either the Fo‐Fo difference map or the correlation matrix, a resampling approach can be employed to allow analysis of clustering between timepoints. This analysis aids in determining the significance of small structural changes by distinguishing inter‐batch variation and conformational sampling within individual states and functionally significant changes across timepoints. This approach was used in Hill *et al*. [38] to ascertain the significance of light induced structural changes in human gamma‐D crystallin.

#### Binning

Data should be split into evenly sampled bins across each dataset ($t_{0}\ldots t_{n}$), with enough measurements per bin to process a complete structure. There are several ways to split the data:

In *CrystFEL* the ‐‐custom‐split flag allows the data to be split into bins at the merging step using *partialator*. This has the advantage that all data are all indexed, integrated and scaled at the same time.


 $ partialator --custom-split bins.txt





The bins.txt file contains a list of image filenames and paths (commonly.h5, .cbf or .nxs), event numbers (detector frames) and dataset names. The files are split per dataset name, for example


 /path/to/data/run000001.h5 tag-0000001 bin_0


 /path/to/data/run000001.h5 tag-0000002 bin_1


 /path/to/data/run000001.h5 tag-0000003 bin_2


 /path/to/data/run000001.h5 tag-0000004 bin_3


 /path/to/data/run000001.h5 tag-0000005 bin_0


 /path/to/data/run000001.h5 tag-0000006 bin_1


 /path/to/data/run000001.h5 tag-0000007 bin_2


 /path/to/data/run000001.h5 tag-0000008 bin_3


 /path/to/data/run000002.h5 tag-0000001 bin_0


 /path/to/data/run000002.h5 tag-0000002 bin_1


 /path/to/data/run000002.h5 tag-0000003 bin_2





The data are thereby assigned by run name (first parameter) and image tag (second parameter) to each bin name (third parameter). Alternatively, in *xia2.ssx*, the dose_series_repeat flag allows splitting of the data sequentially into n bins.


 dose_series_repeat=n





Jackknife sampling and bootstrapping may also be used to define the bins and can be advantageous when the number of indexed patterns is limited [24]. Sampling should not bias the bins. For example, for fixed‐target experiments, bias will be introduced if the first bin contains only images from the edges of chips or images from a single chip.

Clustering analysis of the batched datasets in reciprocal space is then performed using the *cluster4x* protocol described in the correlation section to generate a correlation and SVD plot. Where structural changes between timepoints are subtle, additional clustering in torsion angle space [14] provides greater insight into the significance of these structural changes.

#### RoPE

**Fig. 5:**
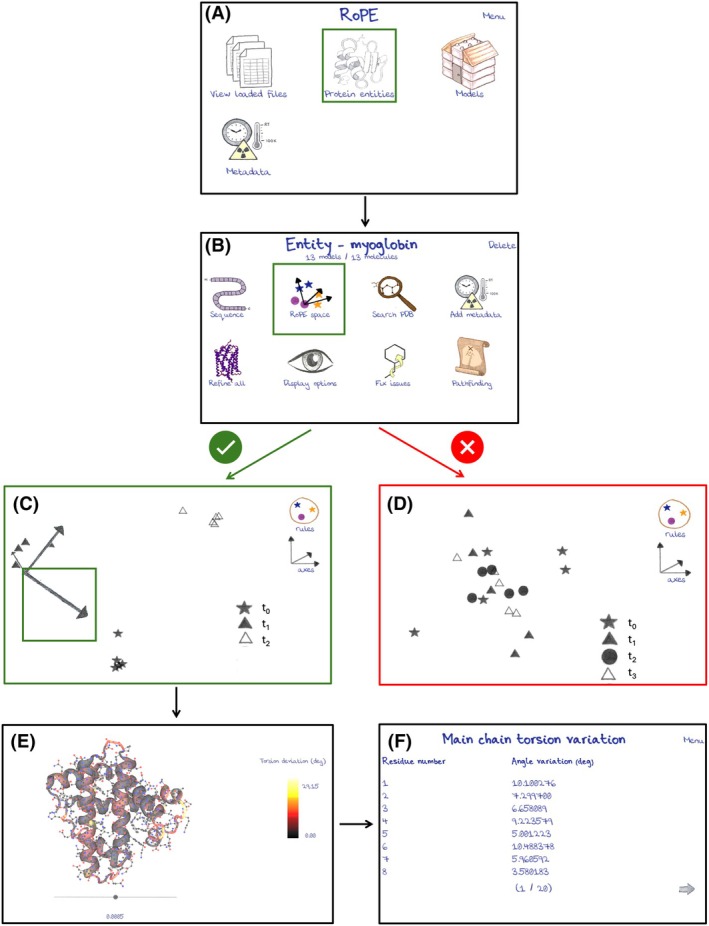
*RoPE* GUI. (A) open *RoPE* in the directory containing refined models, and assign models to a protein entity. (B) refine models and explore SVD results using *RoPE* space. (C) distinct grouping of datasets in *RoPE* space is shown. This is an indicator that the experiment has been successful. (D) no clear trends in clustering manifest as a random distribution of datasets, indicating that the experiment was unsuccessful or there was a problem with data processing. (E) exploring one principal axis visualises structural changes associated with that axis. (F) the list of torsion angle variations along this axis can be output as a CSV file for further analysis. User‐selectable elements are indicated by green rectangles. Data generated for illustrative purposes only.

The *RoPE* software package reduces the model description to torsion angle space and performs a torsion angle refinement followed by PCA to show how proteins cluster in conformational space. Grouping of batches from the same timepoint indicates internal consistency within each timepoint and the software can pull out the structural changes associated with a motion and display them on the reference PDB file. To use *RoPE* for clustering analysis:

1. Run reciprocal space refinement on each of the batched timepoint datasets $t_{0}\ldots t_{n}$. Overfitting (indicated by divergence between $R_{\text{work}}$ and $R_{\text{free}}$ over the course of refinement) is not of the highest concern as long as reciprocal space is largely complete, as the models do not need to be of the highest quality as expected for deposition in order to pull out the significance of differences between timepoints. This should be taken into account when choosing the number of images per batched dataset. The models must all be stored within the same (working) directory.
2. Open the *RoPE* GUI (Fig. 5) and on MacOS X select *find project folder* and select the data directory using the file explorer. On Linux open rope.gui in the working directory.
3. Click on the *models* icon and select *add model*. Add one PDB file as the initialising model. Different chains within the PDB file should be assigned as different entities. This is especially useful where each chain may need to be analysed separately, for example where enzymes have co‐operative active sites or where there are multiple molecules within the asymmetric unit and crystal contacts modulate dynamics/activity.
4. After all entities have been added, return to the Models menu and click on the tools icon to automatically model (auto‐model) all available PDB files. This will automatically match chains present in the PDB files according to the entities, which have been defined in the previous step.
5. Click the back button and select the protein entities icon. Click on the name of the desired protein entity for analysis.
6. Click on the *RoPE* *space* icon and select the number of threads for refinement. After refinement, click *Save and return* to view the clustering results.
7. If timepoints indeed have significant changes between them, the *RoPE* space will show clustering of the batches as shown in Fig. 5 (Step 2). When the experiment may have failed, the batched timepoints will have a tendency to show no overall separation (step 3).
8. Right‐clicking an icon and pressing *set as reference* will display the first three principal component axes. These in turn can be right‐clicked to bring up another menu including the option *explore axis*, which allows the torsion angle changes along the backbone associated with a given principal component to be rendered on the protein. The scale of the changes is shown as the torsion deviation in degrees.

## Tips and tricks

- The *ccp4* task *import_serial* can be used to convert from CIF to MTZ format and to generate data quality reports. This is available through the CCP4i2 GUI [16] or via the command line.

For merged HKL files output from *CrystFEL*, both half datasets, the unit cell file, space group (in Hermann‐Mauguin notation or the space group number) and wavelength (Å) are required variables.ace:


 ccp4-python -m import_serial --hklin data.hkl --half-dataset


 data.hkl1 data.hkl2 --cellfile cell.cell --spacegroup P212121


 --wavelength 1.13 --dmin 1.3





For merged MTZ files output from *xia2* only the merged.mtz and a resolution cut‐off is necessary to output the data quality report.


 ccp4-python -m import_serial --hklin merged.mtz --dmin 1.3

- The current release of *shell_scale* does not apply scaling to uncertainties $\left|\sigma F_{\mathrm{obs}}\right|$. To fix this, edit the file vagabond/shell_scale/main.cpp on line 139, replacing:


 double sigma = mtz->getReal(i, j, k);

with


 double sigma = mtz->getImag(i, j, k);

- *shell_scale* can also be modified to preferentially scale and refine intensities.

To do this, edit vagabond/libsrc/DiffractionMTZ.cpp by adding the lines:


                ampNames.push_back("I");


                ampNames.push_back("IMEAN");





above lines 134–137 and edit vagabond/libsrc/FFT.cpp lines 1408–1409 to:


 colout[4] = MtzAddColumn(mtzout, set, "l", "j");


 colout[5] = MtzAddColumn(mtzout, set, "SIGI", "Q");





Then, from the *Vagabond* main folder rebuild using ninja ‐ C build/current.

- Example *CrystFEL* slurm script for spot‐finding, indexing and integration.


 #!/bin/bash


 #SBATCH -- options for slurm go here


 inp=/path/to/input/bin.lst


 out=/path/to/input/bin.stream


 geom=/path/to/geom/refined.geom


 cell=/path/to/cell/sample.cell


 algor=xgandalf


 peaks=peakfinder8


 thresh=200 #beamline-dependent


 bg_rad=3


 min_snr=4


 int_rad="3,4,5"


 


 module load crystfel


 


 indexamajig -i ${inp} -o {out} -j 48 -g ${geom} -p ${cell}


 --indexing=${algor} --peaks=${peaks} --threshold=${thresh}


 --local -bg -radius=${bg_rad} --min_snr=${min_snr}


 --int -radius=${int_rad}

*CrystFEL* slurm script for scaling and merging.


 #!/bin/bash


 #SBATCH -- options for slurm go here


 input=/path/to/input/bin.stream


 output=bin.hkl


 pointgroup="pointgroup"


 cell=/path/to/cell/sample.cell


 model=unity


 


 module load crystfel


 


 partialator -i ${input} -o ${output} -y ${pointgroup}


 --model=${model} --custom-split bins.txt





Alternatively, an example script is also provided within *CrystFEL* which allows splitting the job over multiple parallel processes: https://gitlab.desy.de/thomas.white/crystfel/‐/blob/master/scripts/turbo‐index‐slurm. Also the *CrystFEL* GUI can be configured to run through slurm.

- Example *DIALS* processing script:


 #! /bin/bash


 #SBATCH --slurm options


 


 


 module load dials


 xia2.ssx


 template=/path/to/data/name_#####.cbf \ #this changes to image


 = for eiger/.h5/.nxs files


 space_group=P212121 \


 unit_cell=a,b,c,alpha,beta,gamma \


 d_min=1.7 \


 spotfinding.max_spot_size=400 \


 spotfinding.min_spot_size=2 \


 #dose_series_repeat=5 \ #auto bins data into timepoints/doses


 #workflow.steps=find_spots+index+integrate #uncomment to set 


 individual steps. Useful for multi chip/column merges


 reference_geometry=/path/to/geometry_refinement/refined.expt


 #comment out to rerefine geometry with dials

where a, b, c are the lengths of the three crystallographic axis in Å and alpha, beta and gamma are the corresponding angles between the axes in degrees. The space group is provided in short space group notation and d_min is the high‐resolution cut‐off in Å.

- The default background in *RoPE* can be changed from *paper* to white by selecting the *menu* then *options* buttons from the start screen.
- *RoPE* can use custom metadata (details in documentation at https://rope.hginn.co.uk/index.php?page=dox) to recolour dataset icons according to continuous discrete variables (e.g. crystallisation conditions).
- Tutorials of the described programs are available online. It is recommended to follow them to further become familiarised with all options and possibilities:
- *Xtrapol8*: https://github.com/ElkeDeZitter/Xtrapol8‐tutorial
- *RoPE*: https://rope.hginn.co.uk/index.php?page=tutorials
- cluster4x: https://www.youtube.com/watch?v=EhqBol‐KYFc
- Multiple strategies exist for depositing structures of intermediate states refined in ESFAs to the Protein Data Bank, and no consensus has yet been reached. The extrapolated structure can be deposited at such, but more preferable might be the deposition of an ensemble with reference and triggered states at their relative occupancies to more accurately reflect the original raw data.
- Software packages are regularly updated. Make sure to use the latest version to keep track of the latest developments and best practices.

## Conflict of interest

The authors declare no conflict of interest.

## Author contributions

JH, YP, EDZ, HMG and BAY all prepared figures and wrote manuscript. JH and YP wrote scripts.

## Data Availability

*Xtrapol8* can be downloaded from https://github.com/ElkeDeZitter/Xtrapol8. *Vagabond* can be downloaded from https://github.com/helenginn/vagabond. *RoPE* can be downloaded from https://github.com/helenginn/rope.
